# Association of uncertain significance genetic variants with myocardial mechanics and morphometrics in patients with nonischemic dilated cardiomyopathy

**DOI:** 10.1186/s12872-024-03888-x

**Published:** 2024-04-25

**Authors:** Karolina Mėlinytė-Ankudavičė, Marius Šukys, Gabrielė Kasputytė, Ričardas Krikštolaitis, Eglė Ereminienė, Grytė Galnaitienė, Vaida Mizarienė, Gintarė Šakalytė, Tomas Krilavičius, Renaldas Jurkevičius

**Affiliations:** 1 Department of Cardiology, Medical Academy, Lithuanian University of Health Sciences, Kaunas, LT-44307 Lithuania; 2 Institute of Cardiology, Lithuanian University of Health Sciences, Kaunas, LT-50162 Lithuania; 3 Department of Genetics and Molecular Medicine, Lithuanian University of Health Sciences, Kaunas, LT-50161 Lithuania; 4 Faculty of Informatics, Vytautas Magnus University, Kaunas, LT-44248 Lithuania; 5 Department of Radiology, Medical Academy, Lithuanian University of Health Sciences, Kaunas, LT-44307 Lithuania

**Keywords:** Dilated cardiomyopathy, Genetics, Heart failure, 2D echocardiography, Magnetic resonance imaging

## Abstract

**Background:**

Careful interpretation of the relation between phenotype changes of the heart and gene variants detected in dilated cardiomyopathy (DCM) is important for patient care and monitoring.

**Objective:**

We sought to assess the association between cardiac-related genes and whole-heart myocardial mechanics or morphometrics in nonischemic dilated cardiomyopathy (NIDCM).

**Methods:**

It was a prospective study consisting of patients with NIDCM. All patients were referred for genetic testing and a genetic analysis was performed using Illumina NextSeq 550 and a commercial gene capture panel of 233 genes *(Systems Genomics, Cardiac-GeneSGKit®).* It was analyzed whether there are significant differences in clinical, two-dimensional (2D) echocardiographic, and magnetic resonance imaging (MRI) parameters between patients with the genes variants and those without. 2D echocardiography and MRI were used to analyze myocardial mechanics and morphometrics.

**Results:**

The study group consisted of 95 patients with NIDCM and the average age was 49.7 ± 10.5. All echocardiographic and MRI parameters of myocardial mechanics (left ventricular ejection fraction 28.4 ± 8.7 and 30.7 ± 11.2, respectively) were reduced and all values of cardiac chambers were increased (left ventricular end-diastolic diameter 64.5 ± 5.9 mm and 69.5 ± 10.7 mm, respectively) in this group. It was noticed that most cases of whole-heart myocardial mechanics and morphometrics differences between patients with and without gene variants were in the genes *GATAD1*, *LOX*, *RASA1*, *KRAS*, and *KRIT1*. These genes have not been previously linked to DCM. It has emerged that *KRAS* and *KRIT1* genes were associated with worse whole-heart mechanics and enlargement of all heart chambers. *GATAD1*, *LOX*, and *RASA1* genes variants showed an association with better cardiac function and morphometrics parameters. It might be that these variants alone do not influence disease development enough to be selective in human evolution.

**Conclusions:**

Combined variants in previously unreported genes related to DCM might play a significant role in affecting clinical, morphometrics, or myocardial mechanics parameters.

**Supplementary Information:**

The online version contains supplementary material available at 10.1186/s12872-024-03888-x.

## Introduction

DCM is characterized as left ventricular (LV) or biventricular systolic dysfunction and dilatation, and there are no abnormal loading conditions or coronary artery disease [1]. The frequency of the disease varies from 1:250 to 1:2.500 and is the most common cause of heart failure (HF) in the young and the leading cause of heart transplantation worldwide [2]. The disease progression is related to various factors, such as toxic damage, delayed initiation of medical treatment, and the presence of an adverse genetic background [3]. Genetic evaluation has become an important integral part of patient care in cardiomyopathies, and can help predict prognosis. There are many genes and alleles related to DCM, so detailed genetic testing encompasses ever-increasing gene panels [4]. The most detected mutations in DCM are truncating variants in *TTN* [4, 5]. However, the spectrum of genes related to DCM is broad, and our knowledge of the natural history of genetic DCM is poor [6]. Failure to find genetic variants does not necessarily mean that there is no genetic etiology to the DCM [7]. Most diagnostic panels contain a variety of genes whose contribution to DCM is not entirely clear. Therefore, newly notified genes have uncertain molecular or clinical significance [8]. The effort to determine a relationship between gene variants of unknown significance and a phenotype of the disease is important not only for cardiovascular research but also for the clinical genetics of patients and their families [9]. Cardiac MRI and echocardiography are the most used imaging methods in the diagnosis of DCM. However, the phenotype of DCM can be diverse, that is why the evaluation of the whole-heart myocardial mechanics and morphometrics is important.

In this work, we aim to evaluate whether the various variants in cardiac-related genes are associated with changes in the phenotype of the whole-heart myocardial mechanics or morphometrics in patients with nonischemic dilated cardiomyopathy (NIDCM).

## Methods

### Study population

It was a prospective study consisting of 95 patients with NIDCM. The NIDCM diagnosis was defined according to the World Health Organization criteria and the latest European Society of Cardiology (ESC) proposal [1, 10].

The study included outpatients and hospitalized patients for the first time diagnosed with NIDCM. The exclusion criteria were ischemic coronary disease (history of myocardial infarction, revascularization, presence of epicardial coronary artery diameter stenosis > 50%), significant valve disease, inflammatory myocardial disease, or kidney disease (eGFR < 30 ml/min/1.73 m2), tachycardia-induced HF, peripartum cardiomyopathy, toxic damage (alcohol, drugs), under the age of 18 and a poor echocardiographic or MRI quality. The ischemic coronary disease was excluded by angiography or computed tomography.

All patients underwent a detailed physical examination, laboratory tests, a 12-lead baseline electrocardiogram (ECG), a 2D transthoracic echocardiography, and 24-hour Holter monitoring. As a part of the protocol, patients were referred for genetic testing and consulted by a geneticist. Ethical approval was obtained for the study by the Kaunas Regional Biomedical Research Ethics Committee, and all participants gave written informed consent before enrollment.

### Genetic analysis

Gene sequencing was performed using Illumina NextSeq 550 and a commercial gene capture panel of 233 genes *(Systems Genomics, Cardiac-GeneSGKit®)* (Table 1S in the Data Supplement). This panel was chosen as it covers all clinically validated cardiomyopathy genes and other genes that might affect the cardiovascular system. All samples reached a sequencing depth of at least 200 of the target regions. Sequencing was repeated if not reach the mentioned depth. Copy number variants were assessed during clinical evaluation, but in this study, they were not included. Target regions were aligned to Grh38.

**Table 1:** Patient’s demographic and clinical characteristics

Demographic and clinical variables	NIDCM group (*n* = 95)
Age, years	49.7 ± 10.5
Male, n (%)	66 (69.5)
BMI, kg/m^2^	29.0 ± 5.8
Systolic blood pressure, mmHg	126.0 ± 13.3
Heart rate, bpm	79.1 ± 17.3
QRS duration, ms	119.1 ± 29.9
NYHA class, n (%) I II III IV	3 (3.2) 25 (26.3) 56 (58.9) 11 (11.6)
VT, n (%)	27 (28.4)
*Cardiovascular risk factors, n (%)*	
Arterial hypertension	36 (37.9)
Dyslipidemia	45 (41.3)
Diabetes	8 (7.3)
Smoker	44 (40.4)
Obesity	70 (64.2)
AF	37 (38.9)
*Pharmacotherapy (at baseline), n (%)*	
ACE-I/ARB	43 (39.4)
Betablocker	51 (46,7)
CCB	28 (25.6)
Aldosterone antagonist	9 (8.2)
Statins	20 (18.3)
Diuretic	3 (2.7)
Laboratory values	
BNP, ng/l	1385.2 ± 986.9
TnI, ng/l	0.2 ± 0.3
hs-CRP, mg/l	3.0 ± 2.7
CRP, mg/l	9.0 ± 5.7
NLR	3.1 ± 3.0
ST2, pg/ml	49.9 ± 80.3

ACE-I -angiotensin-converting enzyme inhibitor; AF - atrial fibrillation; ARB - angiotensin receptor blocker; BMI – body mass index; BNP – brain natriuretic peptide; CRP - C-reactive protein; Hs-CRP -high sensitivity C-reactive protein; LBBB – left bundle branch block; NIDCM - nonischemic dilated cardiomyopathy; NYHA – New York Heart Association; NLR - Neutrophil-to-lymphocyte ratio; ST2 - suppression of tumorigenicity 2 TnI – troponin I; VT - ventricular tachycardia

### Data collection for genetic analysis

This study included information on gene variants, clinical variables, and 2D echocardiography and MRI parameters in 95 patients. It was analyzed whether there are significant differences in clinical, 2D echocardiographic, and MRI parameters between patients with the genes variants and those without. First, it was checked whether the patient had at least one variant of each gene. The distribution of patient samples according to the existence of variants in the respective gene is provided in Table 2S (Data Supplement). Only cases with a sample of at least 20 were selected to compare the presence and absence samples of gene variants. A total of 121 cases were found.

**Table 2:** 2D echocardiographic and MRI characteristics

Parameters NIDCM group (*n* = 95)			
2D echocardiographic		MRI	
LVEDD, mm	64.5 ± 5.9	LVEDD, mm	69.5 ± 10.7
LVESD, mm	55.5 ± 7.5	LVEF, %	30.7 ± 11.2
LVGLS, %	-8.8 ± 2.8	LVEDVi, ml	150.1 ± 42.7
LVGCS, %	-13.4 ± 4.8	LVESVi, ml	106.8 ± 43.6
LVGRS, %	20.9 ± 9.2	RVEDVi, ml	111.6 ± 89.9
LVEF, %	28.4 ± 8.7	RVESVi, ml	55.1 ± 28.8
RVFWLS, %	-18.0 ± 3.2	LVGLS, %	-11.1 ± 4.8
LAVi, ml	53.3 ± 25.4	LVGCS, %	16.9 ± 7.5
LAScd, %	-13.6 ± 4.5	RV strain, %	-15.7 ± 6.5
LASr, %	22.5 ± 7.9	LAS, %	13.1 ± 11.4
LASct, %	-10.0 ± 4.6	LAA	32.9 ± 10.0
RAScd, %	-15.1 ± 5.6	RAS, %	14.8 ± 8.1
RASr, %	28.3 ± 7.1	RAA	29.1 ± 8.3
RASct, %	-12.4 ± 5.2		

LVESD – left ventricular end-systolic diameter; LVEDD – left ventricular end-diastolic diameter; LVEDV – left ventricular end-diastolic volume; LVESV – left ventricular end-systolic volume; LVGLS – left ventricular global longitudinal strain; LVGCS – left ventricular global circumferential strain; LVGRS – left ventricular global radial strain; LVEF – left ventricular ejection fraction; RVEDV – right ventricular end-diastolic volume; RVESV – right ventricular end-systolic volume; RVFWLS – right ventricular free wall longitudinal strain; RV – right ventricular; LAV – left atrial volume; RAV – right atrial volume; LASr – left atrial strain during reservoir phase; LAScd – left atrial strain during conduit phase; LASct – left atrial strain during contraction phase; RAScd – right atrial strain during conduit phase; RASct – right atrial strain during contraction phase; RASr – right atrial strain during reservoir phase; LAS - left atrial strain; RAS - right atrial strain; LAA - left atrial area; RAA - right atrial area; GRV - global regurgitation volume; 2D - two dimensional; MRI - magnetic resonance imaging; NIDCM - nonischemic dilated cardiomyopathy

The genes listed in Table 2S, were tested whether there is a statistically significant difference in the values of clinical, 2D echocardiographic, and MRI parameters between the groups of patients who have the corresponding gene variant and those who do not.

### 2D echocardiographic analysis

**Fig. 1:**
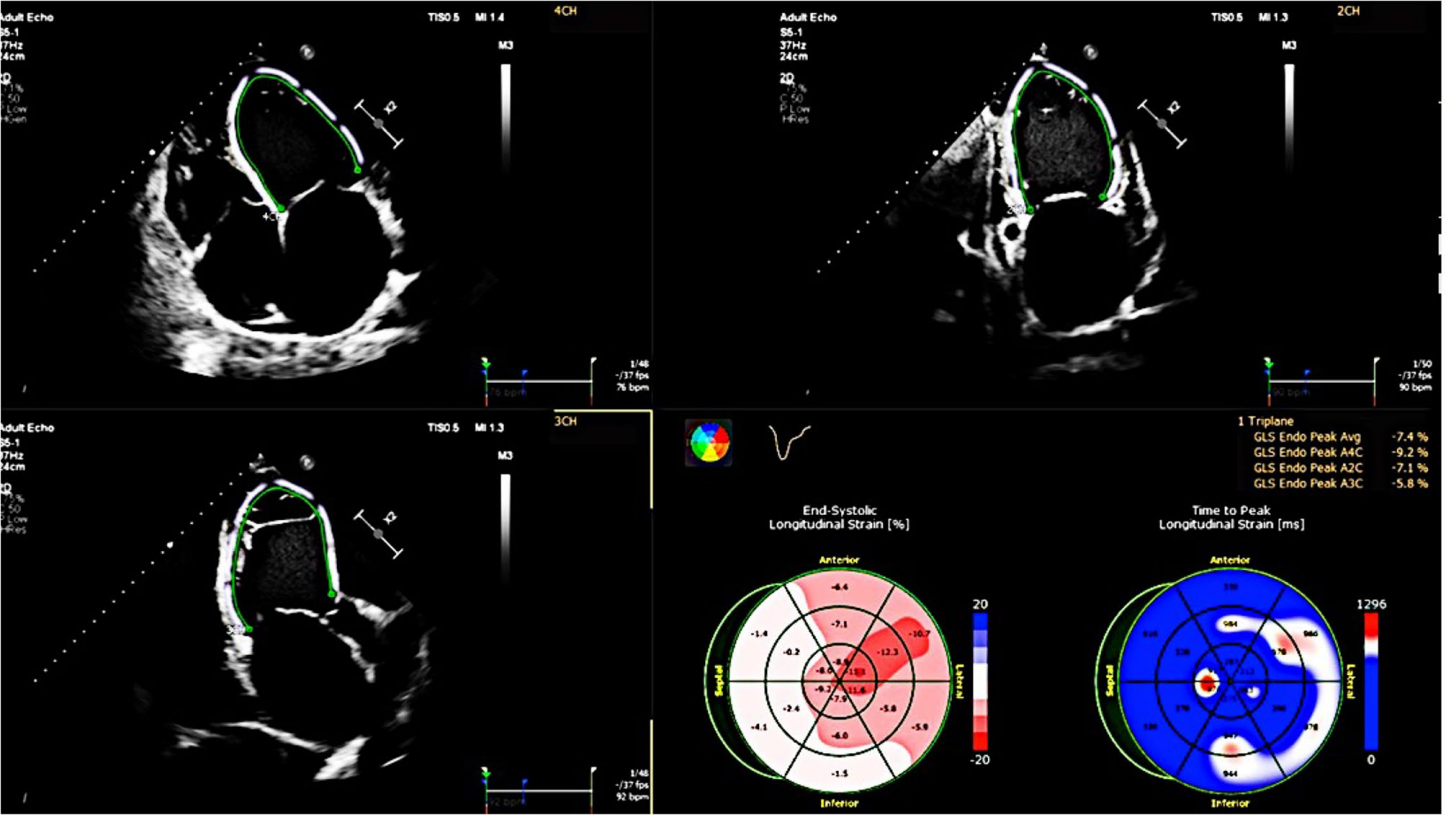
Left ventricular speckle tracking analysis shows decreased GLS strain values using apical four, three, and two-chamber views

2D echocardiography was performed using Philips EPIQ 7 according to a pre-specified protocol with recommendations of the European Association of Cardiovascular Imaging [11]. All images were analyzed using offline TomTec Imaging Systems (Unterschleissheim, Germany). LV end-systolic diameter (LVESD) and end-diastolic diameter (LVEDD) were evaluated from the parasternal LV long-axis view and measured below the level of the mitral valve leaflet tips. LV volumes were calculated by the biplane method of disk summation. Left ventricular ejection fraction (LVEF) was calculated by Simpson’s biplane method. Right ventricular (RV) dimensions were estimated from a RV-focused apical four-chamber view. Left atrial (LA) size was measured at the end of LV systole and LA volume was assessed in apical four- and two-chamber views using the disk summation algorithm. Right atrial (RA) volume was evaluated using a single-view area-length technique [11]. The EACVI/ASE/Industry Task Force consensus document was used to standardize LA, RV, and RA myocardial deformation parameters [12]. The LV global longitudinal strain (LVGLS), global circumferential strain (GCS), and global radial strain (GRS) were defined as the average peak strain values automatically generated from the 16 segmental strain curves by the software [13]. For the assessment of LVGLS, apical four-chamber, two-chamber and long-axis views were acquired (Fig. 1). LV GCS and GRS were measured by endocardial tracing in the basal, middle, and apical levels of LV short-axis views.

**Fig. 2:**
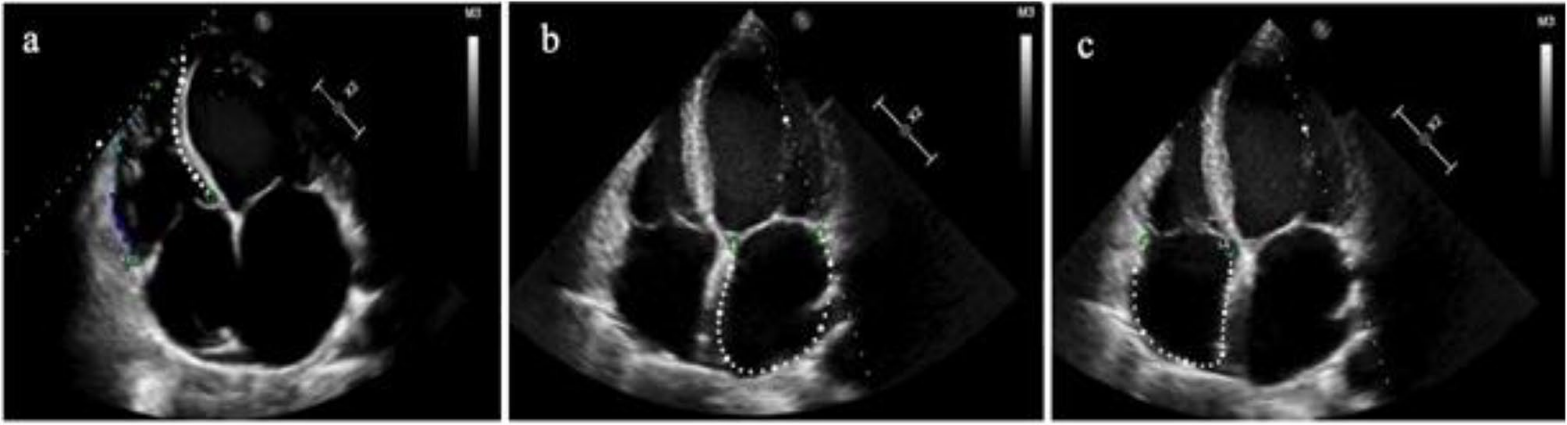
a Right ventricular speckle tracking analysis (RV free wall longitudinal strain is indicated only by a colored dotted line); b-c Lef and right atrial strains are composed of six segments and indicated by a whited dotted line respectively

RV-free wall longitudinal strain (RVFWLS) was calculated from the RV-free wall segments (Fig. 2a). The single apical four-chamber view was used to automatically assess the values for LA strain during the reservoir (LASr), conduit (LAScd), and contraction (LASct) phase Fig. 2b). RA endocardial border was manually traced in a four-chamber view, thus delineating a region of interest, composed of six segments. The reservoir (RASr), conduit (RAScd), and contraction (RASct) phases to evaluate the RA function were analyzed (Fig. 2c).

### MRI protocol and analysis

The MRI examinations were conducted with a 3.0-T MRI scanner (MAGNETOM Skyra, Siemens Healthcare, Erlangen, Germany) using an 18-channel cardiac coil and ECG gating. Cine images were acquired using standard balanced steady-state free precession (bSSFP) sequences in long axes (2-, 3- and 4-chamber) and the stack of short axis (covering entire ventricles) views during an expiratory breath hold. MRI was analyzed by a trained, blinded observer.

**Fig. 3:**
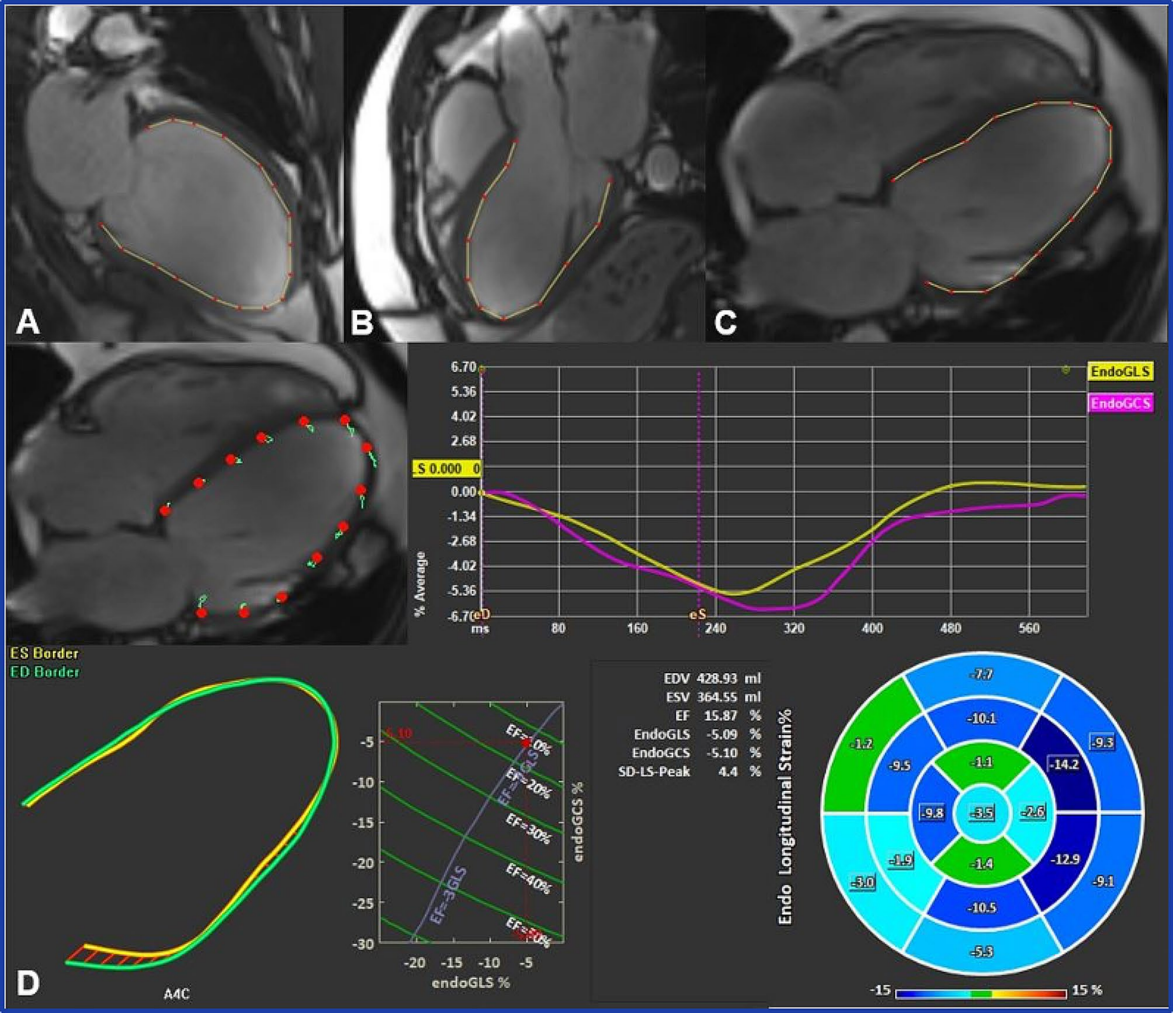
Left ventricular global longitudinal strain assessment using feature tracking software. Endocardial borders were delineated on long-axis two- (**A**), three- (**B**), and four-chamber (**C**) SSFP cine images in end-systole and end-diastole. The final automatic calculation was performed by the software: the average GLS of all 17 cardiac segments in this case was -5.09 (**D**)

**Fig. 4:**
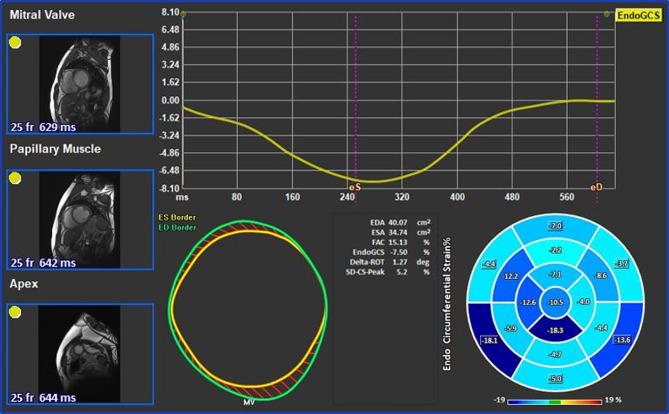
Left ventricular global circumferential strain assessment

Cardiac morphological and functional analysis was performed using the software application QMass (Medis Suite 3.1, Medis Imaging, Leiden, The Netherlands). LV and RV volumes, LVEF, RVEF, and LV mass were evaluated from short-axis cine images using standard volumetric techniques [14]. Endocardial and epicardial borders were drawn manually in end-diastole and end-systole (with papillary muscles included) and the quantification was performed automatically by the software. LVEDD was measured in a short-axis basal plane view. Areas of the left and right atrium (LAA and RAA, respectively) were measured on a long-axis 4-chamber view in the end-systole. MRI strain analysis was performed using the feature tracking application QStrain (Medis Suite 3.1, Medis Imaging, Leiden, The Netherlands). The endocardial borders of all cardiac chambers were traced manually in end-systole and an automated tracking algorithm was applied [15]. Adjustments were made to the contours where needed. Peak systolic and diastolic strain rates were detected and global peak values were calculated by the software. LVGLS (Fig. 3) and GCS (Fig. 4) were derived by averaging the peak strain values of individual segments using a 17-segment model.

The LV GCS was automatically calculated from previously traced points in QMass on short-axis views at the base, mid, and apex planes during end-systole and end-diastole (Fig. 4).

**Fig. 5:**
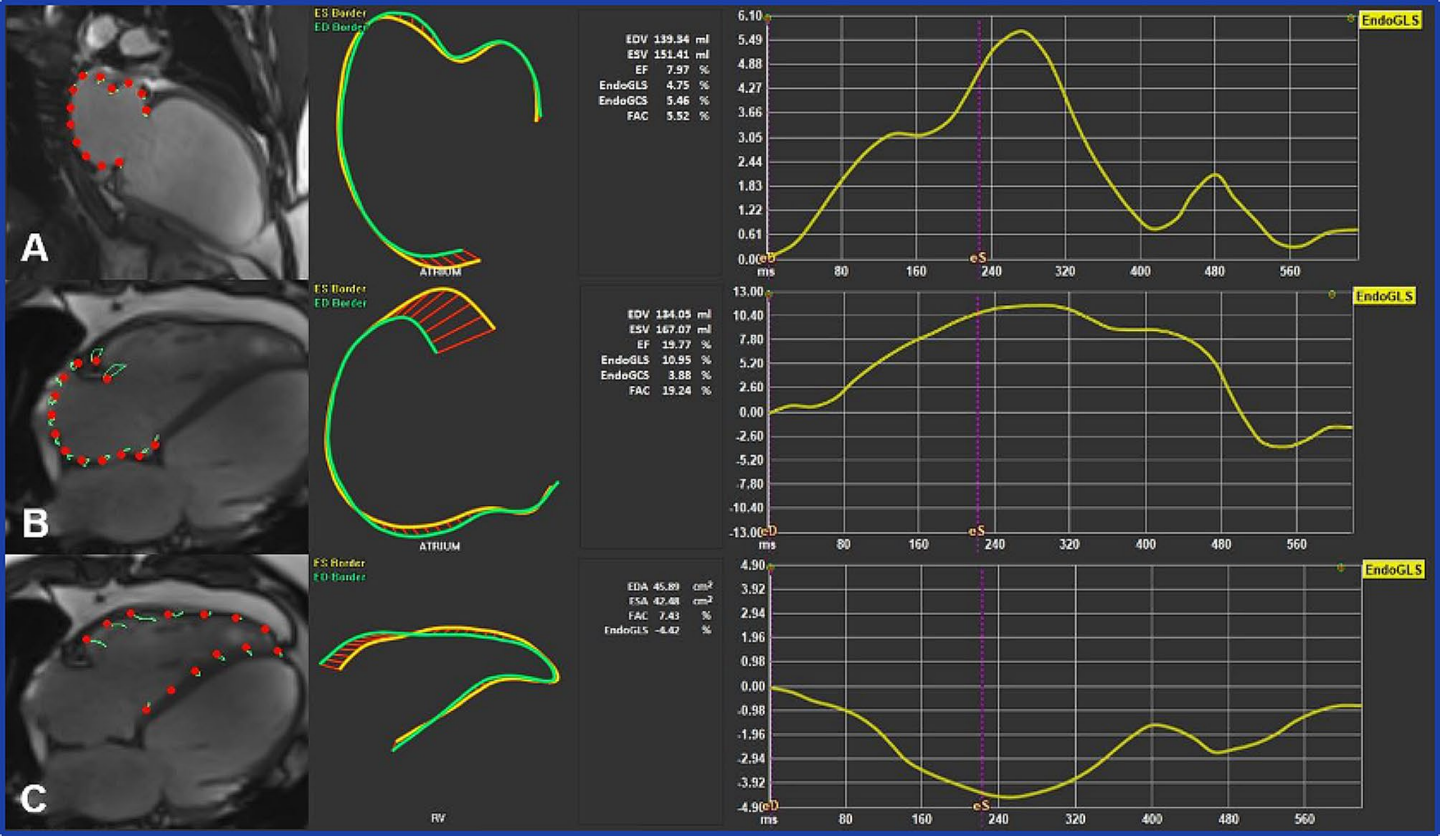
Global longitudinal strain assessment of the left atrium (A), right atrium (B), and right ventricle (C)

RV and RA GLS were calculated from 4-chamber, LA GLS was calculated from 2-chamber long-axis views (Fig. 5).

### Statistical analysis

Different types of statistical tests were used to compare groups. First, the Shapiro-Wilk test was applied to test the normality of continuous quantitative variables. ANOVA was used for normally distributed data, and the Kruskal-Wallis test was used for the variables, which did not meet the normality assumption. The chi-square test was applied for categorical variables. The chi-square test and ANOVA show whether there is a statistically significant difference between the group means, while the Kruskal-Wallis test indicates whether there is a statistically significant difference in medians. Thus, in all cases, when the p-value of the statistical test is less than 0.05 (the corresponding confidence level is 95%), it is assumed that a statistically significant difference is observed between the parameters of patients with and without the analyzed gene variant. Multivariable binary logistic regression analysis was performed to find associations between 2D echocardiographic and MRI variables and gene variants. All univariable associated factors were added to the model, which was improved using a backward stepwise model selection by AIC. Statistical analysis was performed by using RStudio (version 2022.12.0 + 353).

## Results

### Clinical, demographic, and imaging data of the patients

The study group consisted of 95 patients with NIDCM. The average age in the study group was 49.7 ± 10.5 and there were more males (66 (69.5%)). The tendency of the study group to be overweight was observed (body mass index 29.0 ± 5.8). The mean systolic blood pressure and heart rate were within normal limits. Patients with NIDCM had a widened QRS complex. There were more patients with NYHA functional classes II-III (26.3% and 58.9%, respectively). Ventricular tachycardia was present in 28.4% of patients and atrial fibrillation in 38.9%. Each patient with NIDCM had more than one risk factor such as arterial hypertension (AH), dyslipidemia, diabetes, smoking, or obesity. During the initial contact, the indicated drugs were usually used for AH - alone or in combination therapy. The biomarkers of HF (troponin I (TnI), B-type natriuretic peptide (BNP), high-sensitivity C-reactive protein (hs-CRP), suppression of tumorigenicity 2 (ST2), and neutrophil-to-lymphocyte ratio (NLR)) were elevated (Table 1).

The patients with NIDCM had dilated both ventricles and atria. Measurements of the diameters and volumes were made using 2D echocardiography and MRI. A significant reduction of LVEF was detected by both methods. All myocardial mechanical parameters of both ventricles and atria were reduced. There are no cut-off limits for GRV severity, however, our study revealed that the mean of GRV was increased (45.8 ± 33.3) (Table 2).

### Genetic analysis

Genes with the most cases of statistically significant differences in clinical, 2D echocardiographic, and MRI parameters between patients with and without gene variants were selected. The largest differences in total cardiac myocardial mechanics and morphometry between patients with and without gene variants were observed in the genes *KRAS, KRIT1, GATAD1, LOX* and *RASA1*. Only those parameters with which the listed genes had significant associations are presented.

It has been observed that the influence of determined gene mutations can be twofold: the values of the clinical, 2D echocardiographic, and MRI parameters are better or worse when the existence of a gene variant is observed or not. The median values and standard deviations of the mentioned parameters with statistically significant differences between the groups of patients with and without the gene variants are presented in Tables 3 and 4. Table 3 shows the cases associated with worse parameters with *KRAS* and *KRIT1* gene variants. These gene variants in patients with NIDCM were related to enlargement of both ventricles and atria and worse RV function.

**Table 3:** Genes associated with worse clinical, 2D echocardiographic and MRI parameters

	KRAS				KRIT1		
	No gene variants	Gene variants	p- value		No gene variants	Gene variants	p- value
2D echocardiographic parameters				2D echocardiographic parameters			
LVEDD, mm	62 ± 5.65	64.5 ± 6.39	0.041	RVEDV, ml	131.14 ± 40.23	174 ± 50.96	0.002
LVESV, ml	138 ± 63.71	161.5 ± 55.02	0.043	RVEDVi, ml/2	61.86 ± 19.80	76.99 ± 25.22	0.004
MRI parameters				MRI parameters			
RVEF, %	43.5 ± 6.96	40 ± 10.45	0.023	RVEF, %	43.48 ± 7.42	38 ± 9.93	0.037
LVEDD, mm	69 ± 6.70	75 ± 17.30	0.003	RVESV, ml	96 ± 54.14	117 ± 67.26	0.022
LVEDV, ml	272 ± 76.66	356 ± 92.29	0	RVESVi, ml/m2	36.85 ± 15.04	45.8 ± 21.95	0.001
LVEDVi, ml/m2	135.0 ± 40.36	174.6 ± 39.46	0	LVEDV, ml	290 ± 75.73	377 ± 109.77	0.038
LVESV , ml	186 ± 78.84	274 ± 90.67	0.001	RVEDV, ml	192.5 ± 56.16	228 ± 57.49	0.004
LVESVi, ml/m2	90.0 ± 42.31	126.4 ± 39.50	0.001	RAA, cm2	26.7 ± 8.52	31 ± 8.76	0.006
RVEDV, ml	188 ± 328.56	219 ± 67.57	0.033				
LAA, cm2	30.5 ± 9.55	36.5 ± 9.99	0.009				

2D – two dimensional; LAA – left atrial area; LVEDD – left ventricular end-diastolic diameter; LVEDV – left ventricular end-diastolic volume; LVEDVi – left ventricular end-diastolic volume index; LVESV – left ventricular end-systolic volume; LVESVi – left ventricular end-systolic volume index; LVGLS – left ventricular global longitudinal strain; LVGCS – left ventricular global circumferential strain; MRI - magnetic resonance imaging; RA – right atrial; RAA - right atrial area; RVEF – right ventricular ejection fraction; RVEDV – right ventricular end-diastolic volume; RVESV – right ventricular end-systolic volume; RVESVi – right ventricular end-systolic volume index

Table 4 identifies those cases that are associated with better 2D echocardiographic and MRI parameters in the presence of a gene variant in the study group. *GATAD1, LOX*, and *RASA1* gene variants in patients with NIDCM were related to smaller size of both ventricles and atria and better cardiac function parameters.

**Table 4:** Genes associated with better clinical, 2D echocardiographic and MRI parameters

	GATAD1				LOX				RASA1		
	No gene variants	Gene variants	p-value		No gene variants	Gene variants	p-value		No gene variants	Gene variants	p-value
Clinical parameters				Clinical parameters				2D echocardiographic parameters			
BNP, ng/l	586.4 ± 1446.73	176 ± 1161.97	0.009	BNP, ng/l	622 ± 1272.34	246.2 ± 1564.83	0.026	RAV, ml	76.9 ± 27.56	70.3 ± 17.19	0.03
2D echocardiographic parameters				2D echocardiographic parameters				RVEDV, ml	165 ± 51.85	129.7 ± 36.82	0.026
LAVi, ml/m2	50.4 ± 26.04	41.7 ± 22.93	0.027	LVEDVm ml	232 ± 82.59	203 ± 43.03	0.033	MRI parameters			
GRV, ml	61.5 ± 29.67	0 ± 37.95	0.004	LVESV, ml	164 ± 68.35	131.5 ± 40.38	0.013	LVESV, ml	210 ± 92.18	193.5 ± 79.65	0.041
LVEDV, ml	220.9 ± 75.71	189 ± 60.73	0.039	LVESVi, ml/m2	74.2 ± 37.91	60.4 ± 19.88	0.033	RVEDV, ml	211 ± 380.84	183 ± 51.52	0.01
LVEDVi, ml/m2	111.8 ± 37.59	100.5 ± 29.79	0.035	RVEDV, ml	158 ± 48.04	126.7 ± 37.75	0.006	RVESV, ml	128 ± 62.94	89.5 ± 46.56	0.001
LVESV, ml	161.5 ± 63.97	112 ± 47.44	0.007	RVEDVi, ml	74.3 ± 24.30	58.1 ± 16.52	0.013	RAA, cm2	30.7 ± 8.69	25.7 ± 8.45	0.006
LVESVi, ml/m2	75.5 ± 35.23	59.4 ± 22.98	0.004	RVESV, ml	93 ± 39.89	66.8 ± 29.11	0.002	LVEF, %	25.7 ± 11.31	34.7 ± 10.28	0.011
LVESDi, ml/m2	27.3 ± 4.21	25 ± 4.01	0.033	RVESVi, ml/m2	43.7 ± 20.02	35.5 ± 13.14	0.002	LVGLS,%	-9.0 ± 4.62	-13.3 ± 4.50	0.002
LVEF, %	27 ± 8.85	32 ± 7.22	0.012	MRI parameters				LA strain, %	-8.5 ± 7.23	-13.4 ± 14.40	0.027
LVGLS, %	-8.1 ± 2.64	-10.5 ± 3.10	0.002	LVEDV, ml	302.5 ± 92.40	254 ± 72.96	0.04	RV strain, %	-12.7 ± 6.71	-16.7 ± 5.44	0.002
MRI parameters				LVEDVi, ml/m2	146.03 ± 42.82	127.3 ± 39.69	0.027	RA strain%	-12.7 ± 6.75	-16.1 ± 8.95	0.007
LVGLS, %	-10.0 ± 4.78	-12.2 ± 4.36	0.019	RVEDV, ml/m2	208.5 ± 341.87	176 ± 40.64	0.014				
LVGCS, %	-15.2 ± 7.22	-18.9 ± 7.55	0.014								

LVEDD – left ventricular end–diastolic diameter; LVESV – left ventricular end–systolic volume; RVEF – right ventricular ejection fraction; LVEDV – left ventricular end–diastolic volume; LVEDVi – left ventricular end–diastolic volume index; LVESV – left ventricular end–systolic volume; LVESVi – left ventricular end–systolic volume index; RVEDV – right ventricular end–diastolic volume; LAA – left atrial area; RVEDVi – right ventricular end–diastolic volume index; RVESV – right ventricular end–systolic volume; RVESVi – right ventricular end–systolic volume index; RAA – right atrial area; 2D – two dimensional; MRI – magnetic resonance imaging

2D – two dimensional; BNP – B-type natriuretic peptide; GRV – global regurgitation volume; LA – left atrial; LAA – left atrial area; LAVi – left atrial volume index; LVEDD – left ventricular end-diastolic diameter; LVESDi – left ventricular end-systolic diameter index; LVEDV – left ventricular end-diastolic volume; LVEDVi – left ventricular end-diastolic volume index; LVEF – left ventricular ejection fraction; LVESV – left ventricular end-systolic volume; LVESVi – left ventricular end-systolic volume index; LVGLS – left ventricular global longitudinal strain; LVGCS – left ventricular global circumferential strain; MRI - magnetic resonance imaging; RA – right atrial; RAA - right atrial area; RV – right ventricle; RVEDV – right ventricular end-diastolic volume; RVEDVi – right ventricular end-diastolic volume index; RVESV – right ventricular end-systolic volume; RVESVi – right ventricular end-systolic volume index.

**Fig. 6:**
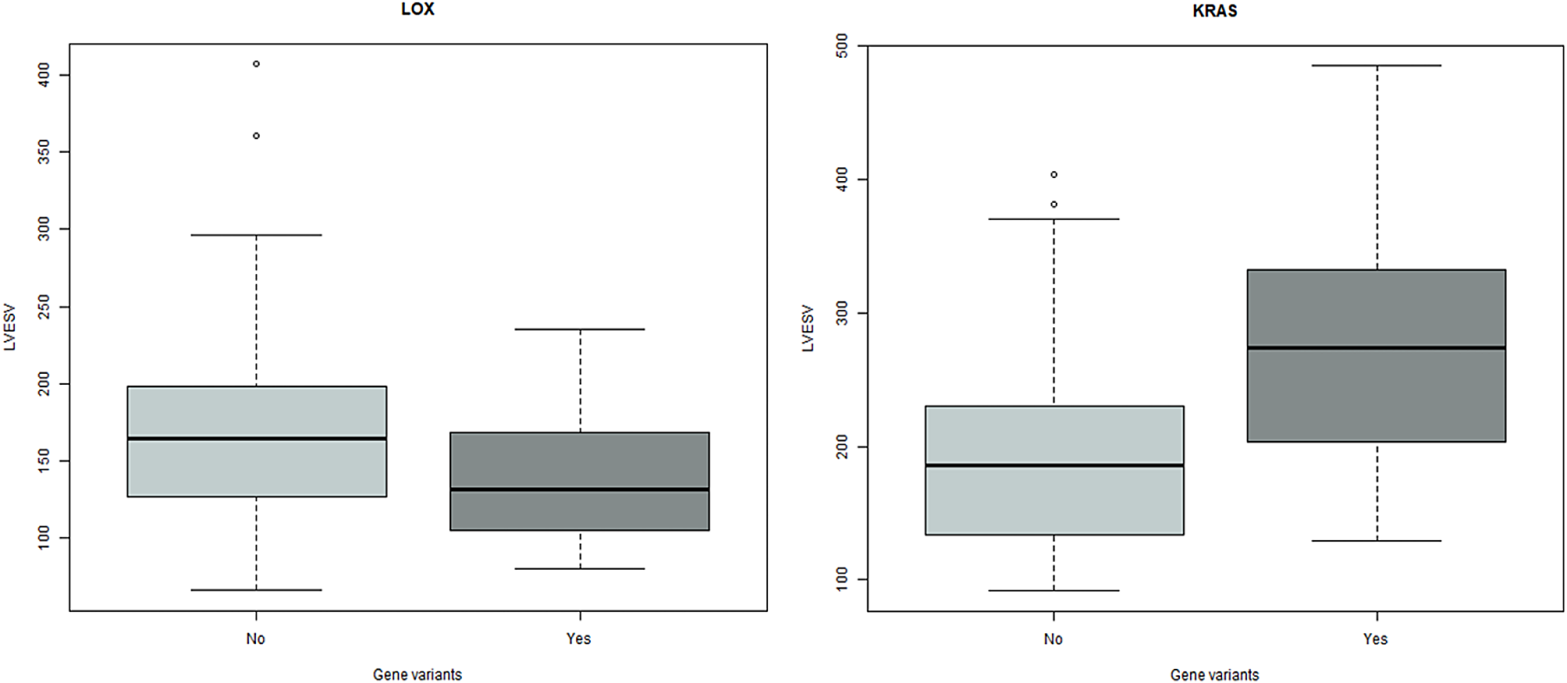
The example of LVESV size distribution based on gene variants

The box plots in Fig. 6 show the example of LVESV distribution and skewness based on the groups of patients with and without the gene variants and consist of the minimum and maximum range values, the upper and lower quartiles, and the median. It was observed that patients with *KRAS* gene variants tend to have higher values of LVESV while patients with the gene variants of *LOX (or GATAD1/RASA1)* have lower values of LVESV.

The results of binary logistic regression analysis showed that LV volumes and RV systolic function were independently associated with the detection of *KRAS/KRIT1* gene variants. (Table 5).

**Table 5:** KRAS/KRIT1 genes variant association with 2D echocardiographic and MRI parameters at univariate and multivariate logistic regression analysis

Variable	Univariate analysis:			Multivariate analysis			
	Odds ratio	95% confidence interval	p-value	Odds ratio	95% confidence interval	p-value	Wald score
2D echocardiographic parameters							
RVESV	1.015	1.003–1.028	0.018*	1.056	0.998–1.124	0.068	3.3
RVESVi	1.025	1.002–1.051	0.041*	0.909	0.805–1.015	0.099	2.7
RVEDV	1.01	1-1.019	0.047*	-	-	-	-
RVEDVi	1.016	0.997–1.036	0.103	-	-	-	-
LVEDD	1.048	0.979–1.126	0.185	-	-	-	-
LVESV	1.004	0.998–1.011	0.211	-	-	-	-
MRI parameters							
LVEDV	1.009	1.003–1.015	0.003*	-	-	-	-
LVEDVi	1.015	1.004–1.028	0.012*	1.06	1.017–1.109	0.008**	6.9
RVEDV	1.01	1.002–1.019	0.015*	1.005	1-1.01	0.304	1.1
LVESV	1.007	1.001–1.012	0.016*	-	-	-	-
RVEF	0.942	0.889–0.991	0.028*	0.914	0.834–0.988	0.035*	4.4
LVESVi	1.011	1.001–1.022	0.046*	0.953	0.913–0.992	0.024*	5.1
LAA	1.044	0.998–1.096	0.072	-	-	-	-
RVESV	1.006	0.999–1.015	0.105	-	-	-	-
RAA	1.038	0.988–1.095	0.149	-	-	-	-
LVEDD	1.014	0.973–1.067	0.526	-	-	-	-

2D - two dimensional; LAA - left atrial area; LVEDD – left ventricular end-diastolic diameter; LVEDV – left ventricular end-diastolic volume; LVEDVi – left ventricular end-diastolic volume index; LVESV – left ventricular end-systolic volume; LVESVi – left ventricular end-systolic volume index; MRI - magnetic resonance imaging; RAA - right atrial area; RVEDV – right ventricular end-diastolic volume; RVEDVi – right ventricular end-diastolic volume index; RVEF – right ventricular ejection fraction; RVESV – right ventricular end-systolic volume; RVESVi – right ventricular end-systolic volume index

## Discussion

Our research findings have unveiled potential links between variations in specific cardiac genes and cardiac function or morphometrics parameters among individuals with DCM. It was revealed that five genes appear to have an impact on myocardial mechanics and morphometrics in an indirect manner and have not been previously linked to DCM. These findings differ from the conventional Genome-wide Association Studies (GWAS), making direct comparisons with other research challenging.

We did not observe significant associations with classical cardiomyopathy genes. This is particularly unexpected considering that variants in genes encoding sarcomere proteins would seemingly exert the greatest influence on the clinical progression of the condition. Most of these genes, such as TTN gene, are rather large and accumulated a substantial amount of mutations during evolution, mostly in non-conserved regions. It is plausible that these mutations elicit minimal adverse effects.

Moreover, *LOX*, *GATAD1*, and *RASA1* genes variants were related to better cardiac function and morphometrics parameters, which might be unexpected because mutations with worse clinical outcomes should be less common in the population. It might be that these variants alone do not influence disease development enough to be selective in human evolution.

Furthermore, lysyl oxidase (LOX) is a protein that includes a family of five copper-dependent enzymes (LOX and four LOX-like isoenzymes (LOXL1–4)) essential for extracellular matrix homeostasis and remodeling. LOX and LOXLs isoenzymes play an important role in the control of vascular homeostasis, remodeling, control of vascular stiffness, oxidative stress, and calcification [16] as well as for the biogenesis of connective tissue matrices [17]. Experimental models have described evidence of the disturbance of LOX/LOXLs activity and cardiovascular diseases [18, 19]. Knock-out animal models have shown a relationship between changes in LOX gene and human aortic aneurysms and dissection [20]. Moreover, LOX family proteins are associated with myocardial stiffness and disturbed LV function. LOX has been observed to be involved in the fibrosis that leads to end-stage DCM. Upregulation of LOX may lead to an imbalance of extracellular matrix degradation and synthesis, so it may participate in DCM and HF remodeling [21]. Variants that decrease this upregulation might be protective. In our study 34 patients had predicted benign variant rs1800449 with frequency in gnomAD of 0,17. Previously, the variant was studied numerous times in association with various cancers and only once in association with cardiovascular disease—higher prevalence for ischaemic heart disease [22]. Other variant was rs368947781 in only 2 patients (gnomAD 0,0001), no studies reported in the literature. Our results revealed the association of the *LOX* gene variants with lesser myocardial damage. Variants in the *LOX* gene were related to smaller ventricles volumes evaluated by 2D echocardiography or MRI. BNP concentration was also lower in cases with *LOX* gene variants.

Our study results showed that the existence of the *GATAD1* gene was related to the better function of both ventricles and smaller dimensions of LV and LA. BNP concentration was also lower in patients with this gene. The *GATAD1* was first described as an ocular development-associated gene in 2002 [23], which, as the name suggests, was studied in the association of eyes development, and in 2011 has been reported in one study as a possible cause for autosomal recessive DCM [24]. Zebrafish knockout models developed phenotypes similar to HF in aged models or after induced stress [25]. In our study, we found four different *GATAD1* gene variants, all reported in ClinVar as benign: rs10281879 (gnomAD 0,11; *n* = 21, 2 homozygous cases) missense variant G54S reported in various associations; rs564747350 (gnomAD 0,0001; *n* = 1) missense variant A202T with no reports in the literature; rs34768413 (gnomAD 0,017; *n* = 2) missense variant R233W; rs139637606 (gnomAD 0,003; *n* = 2). The function of this gene and its product is not well studied, so it is difficult to speculate about the implication of this gene in the development or modification of DCM.

*RASA1* gene is described as associated with vascular malformation syndromes such as Klippel-Trenaunay-Weber syndrome, Sturgeon-Weber syndrome, vein of Galen aneurysmal malformation, etc. *RASA1* is a cytoplasm protein transported to the cell membrane upon increased intracellular Ca2 + concentrations. It participates in cell growth, proliferation, differentiation, and apoptosis. Naturally, this gene is extensively investigated in cancer development [26]. Haploinsufficiency of *RASA1* increases in RAS-MAPK pathway signaling. The same pathway activation is a known mechanism for various RASopathies like Noonan, Costello, and other syndromes. These syndromes have a broad phenotypic spectrum, and the common manifestation is hypertrophic cardiomyopathy [27]. The number of published cases is relatively small, so the wider phenotypic spectrum caused by variants in the *RASA1* gene remains unknown [28]. In our study, the *RASA1* gene was related to the better function and smaller size of both ventricles and better function of atria. We found seven different variants across the *RASA1* gene. The rs111840875 (Ala99Val; gnomAD 0,03; *n* = 7) has several records in ClinVar as benign and no literature reports in cardiovascular association. One record in ClinVar as a variant of unknown significance, and one report in literature in the case of capillary malformation-arteriovenous malformation [28] were detected with variant rs373892264 (Gly156Val; gnomAD 0,03 < 0,01; *n* = 1). Another variant was rs60835975 (gnomAD 0,03; *n* = 10 cases), and one of them is in a homozygous state. The T nucleotide deletion variant is in the homopolymer region just before 11 exons with reports in ClinVar as benign. Other 4 variants are related to homopolymer region just before 14 exon: variant rs377722838 (gnomAD 0,12; *n* = 22) and single cases of rs75512926 (gnomAD 0,02; *n* = 1); rs747412034 (gnomAD 0,003; *n* = 1) and 7 cases rs36000817 (gnomAD 0,064; *n* = 7) with reports in ClinVar as bening and no reports in literature. These variants before 14 exons might be errors due to alignment, as these variants do not repeat in the same cases and are the same variation at the homopolymeric region. More studies are needed to evaluate the association of this gene with DCM.

The Ras family of small G proteins is composed of enzymes that hydrolyze GTP into GDP and is an important component of intracellular signal transduction. Three main human Ras genes are known: H-Ras, K-Ras, and N-Ras. Only a few studies have investigated the role of K-Ras in the heart, and it is related to cardiac cell proliferation [29]. No previous data report *KRAS* gene relationships with DCM pathogenesis. A lot of genes are offered from multiple commercial testing laboratories for the evaluation of DCM. However, *KRAS* is one of the genes that activates the previously mentioned RAS-MAPK pathway and plays an important role in syndromic cardiomyopathy, such as Noonan syndrome, neuromuscular disease, and mitochondrial myopathies [4]. In the *KRAS* gene, we found a single variant rs1137282 c.519T > C, Asp173 = (gnomAD 0,19; *n* = 28). This variant is often reported as benign in ClinVar and is extensively studied in cancer development. One study shows that this variant might increase the gene’s expressivity [30], but it was tested in specific ethnic cell lines and might not be applicable in the European population. In our study, the presence of the *KRAS* gene variants was associated with worse ventricular or atria function or dilatation of cardiac chambers.

*KRIT1* gene germline pathogenic deleterious variants are causative for cerebral cavernous malformations [31]. This gene belongs to the Ras family and regulates endothelial cell junction integrity, stabilizes cell-to-cell junctions, and participates in cell adhesion and migration. The loss of its function leads to increased beta-catenin signaling and abnormal vascular development [32]. It is not clear whether variants of this gene could affect the manifestation of cardiomyopathies. However, we found 4 synonymous variants across our cases: rs149437256 (gnomAD 0,005; *n* = 1); rs11542682 (gnomAD 0,09; *n* = 24); rs143710815 (gnomAD 0,0037; *n* = 1); rs200684252 (gnomAD < 0,001; *n* = 1). All of them are recorded in ClinVar as benign. As in the case of the *KRAS* gene, the presence of the *KRIT1* gene was associated with the enlargement of both ventricles.

Our findings are difficult to compare to other studies because we attempted to analyze all gene variants at simultaneously. Related studies with larger sample sizes can pick up single variants as better predictors for DCM. GWAS with 2719 cases found variants in *SLC6A6*, *BAG3*, and *HSPB7* genes [33]. Other studies found different genes, such as *HCG22*, *ZBTB17*, *FRMD4B*, *USP3*, *TTN*, *SLC39A8*, *MLIP*, *FLNC*, *ALPK3*, and *FHOD3* [34–36]. Many of these studies use a p-value of 5*10^− 8^, which allows us to find a stronger linkage, but many studies have different results, only repeating genes are *HCG22, BAG3*, and *HSPB7*. Applying new techniques, such as long-read sequences, allows alignment to more complete human genomes [37] and having more uniform biobanks with control cases will allow us to better understand the heterogeneity of DCM.

## Limitations

As we combine all variants from panel genes, there may be problems when measuring variants with positive and negative effects together, reducing statistical power. We did not evaluate specific variant effects, so conversely, we cannot say which variant has better or worse outcomes for patients. Nevertheless, we have found cases where we got statistically significant changes. It is not enough to conclude that variants in these genes have a definite impact on the course of the disease, but enough to suspect such linkage and to plan additional studies with larger cases. Our sample size is rather small, which also could affect our findings. Moreover, we do not have healthy controls to compare the frequency of our variants; we can only use gnomAD or similar data, as we lack our national genomic variant database.

## Future directions

We hope, that our work will empower researchers and clinicians in comprehensively analyzing genetic variants, understanding their implications in cardiomyopathies, and potentially paving the way for more effective treatments and personalized healthcare interventions. Based on this work we expect that it will be possible to create a digital database of patients diagnosed with established cardiomyopathies who have undergone genetic panel testing. Also, to perform a comparative analysis of the frequencies of cardiac gene variants with available resources from European or other ethnically similar populations. Explore disease associations linked to the identified genetic variant clusters. Fulfilling these tasks will contribute to a better knowledge of cardiomyopathies development mechanism, genetic causes, and risks. Future scientific studies could be based on further delineating genetic variants in the database and their interactions with the phenotype.

## Conclusions

We found that combined variants in previously unreported genes related to cardiomyopathy might play a significant role in affecting clinical, morphometrics, or myocardial mechanics parameters. Further studies or larger sample sizes are needed to prove such a concept, as with larger cohorts more variation could be detected. The same could be expanded to larger sequencing panels such as whole exomes or whole genomes. Many of the common variants in the population lie in non-coding regions that are poorly covered with panels, so our study focuses more on protein-coding regions where we can expect a better explanation of the significant difference in variant dispersion found.

### Electronic supplementary material

Below is the link to the electronic supplementary material.

Supplementary Material 1

Supplementary Material 2

## Data Availability

The datasets used and/or analyzed during the current study are available from the corresponding author on reasonable request.
