# Supplementary material for: Association of uncertain significance genetic variants with myocardial mechanics and morphometrics in patients with nonischemic dilated cardiomyopathy
Source: BMC Cardiovasc Disord. 2024 Apr 25;24:224. doi: 10.1186/s12872-024-03888-x (PMC11044472; doi:10.1186/s12872-024-03888-x)
Supplement: Supplementary file 2 — Supplementary Material 2 [file 12872_2024_3888_MOESM2_ESM.doc]

Table 2S. Distribution of variants in the respective gene

|  | ***Sample size*** | |  | ***Sample size*** | |  | ***Sample size*** | |
| --- | --- | --- | --- | --- | --- | --- | --- | --- |
| ***Gene*** | ***Gene variants*** | ***No gene variants*** | ***Gene*** | ***Gene variants*** | ***No gene variants*** | ***Gene*** | ***Gene variants*** | ***No gene variants*** |
| *ACTA2* | *25* | *70* | *HRAS* | *61* | *34* | *PNMT* | *53* | *42* |
| *ACTC1* | *51* | *44* | *HRC* | *28* | *67* | *PRKAG2* | *20* | *75* |
| *ACVRL1* | *68* | *27* | *HSPB2* | *21* | *74* | *PRKG1* | *58* | *37* |
| *AIFM3* | *25* | *70* | *IL17RC* | *58* | *37* | *PROS1* | *69* | *26* |
| *ARMS2* | *21* | *74* | *ILK* | *70* | *25* | *PSEN1* | *71* | *24* |
| *BMPR2* | *25* | *70* | *KCND3* | *44* | *51* | *RASA1* | *46* | *49* |
| *BRAF* | *38* | *57* | *KCNE1* | *32* | *63* | *RASA2* | *59* | *36* |
| *CAV3* | *69* | *26* | *KCNE5* | *23* | *72* | *RNA5SP206* | *54* | *41* |
| *CBL* | *70* | *25* | *KCNH2* | *74* | *21* | *RNA5SP280* | *52* | *43* |
| *CCDC40* | *51* | *44* | *KCNJ2* | *25* | *70* | *RPAP2* | *45* | *50* |
| *CHD7* | *23* | *72* | *KCNK16* | *48* | *47* | *RPSA* | *68* | *27* |
| *CHST14* | *26* | *69* | *KCNK17* | *73* | *22* | *RRP8* | *29* | *66* |
| *CITED2* | *46* | *49* | *KCNQ1* | *63* | *32* | *SCN1B* | *63* | *32* |
| *COL4A2* | *49* | *46* | *KHDC4* | *46* | *49* | *SCN2B* | *60* | *35* |
| *COX6CP18* | *27* | *68* | *KRAS* | *28* | *67* | *SGCD* | *70* | *25* |
| *CRYAB* | *31* | *64* | *KRIT1* | *27* | *68* | *SLC2A10* | *71* | *24* |
| *DNAAF3* | *26* | *69* | *LAMP2* | *50* | *45* | *SLMAP* | *72* | *23* |
| *DNASE1L1* | *62* | *33* | *LDB3* | *21* | *74* | *SMAD6* | *22* | *73* |
| *DSC2* | *51* | *44* | *LINC01475* | *47* | *48* | *SNORA6* | *42* | *53* |
| *DTNA* | *66* | *29* | *LMNA* | *51* | *44* | *SNORA62* | *42* | *53* |
| *ENG* | *39* | *56* | *LOX* | *34* | *61* | *SNRNP48* | *51* | *44* |
| *ENPP1* | *53* | *42* | *LRRC56* | *37* | *58* | *SPC24* | *33* | *62* |
| *ETFRF1* | *23* | *72* | *MADD* | *33* | *62* | *SPI1* | *55* | *40* |
| *EYA4* | *57* | *38* | *MAP2K1* | *26* | *69* | *STAMBPL1* | *23* | *72* |
| *FHL1* | *60* | *35* | *MFAP5* | *70* | *25* | *STARD3* | *53* | *42* |
| *FHOD3* | *70* | *25* | *MIR208A* | *46* | *49* | *TAF10* | *64* | *31* |
| *FKRP* | *29* | *66* | *MIR208B* | *31* | *64* | *TAZ* | *69* | *26* |
| *FKTN* | *57* | *38* | *MIR3606* | *48* | *47* | *TBX1* | *67* | *28* |
| *FLNA* | *61* | *34* | *MIR4673* | *58* | *37* | *TBX20* | *66* | *29* |
| *FOXC1* | *40* | *55* | *MIR4783* | *49* | *46* | *TBX4* | *75* | *20* |
| *FOXCUT* | *21* | *74* | *MIR6795* | *59* | *36* | *TFAP2B* | *47* | *48* |
| *GATA4* | *31* | *64* | *MIR6870* | *55* | *40* | *TGFBR2* | *63* | *32* |
| *GATAD1* | *25* | *70* | *MIR6886* | *49* | *46* | *TMEM43* | *47* | *48* |
| *GJA1* | *23* | *72* | *MYLK2* | *23* | *72* | *TNNI3* | *35* | *60* |
| *GLA* | *22* | *73* | *NEXN* | *35* | *60* | *TPP1* | *44* | *51* |
| *GLMN* | *66* | *29* | *NODAL* | *73* | *22* | *TRPM4* | *61* | *34* |
| *GPD1L* | *21* | *74* | *NOTCH2* | *50* | *45* | *UPF1* | *58* | *37* |
| *GUCY1A1* | *71* | *24* | *NPPA* | *28* | *67* | *VANGL1* | *39* | *56* |
| *GYG1* | *45* | *50* | *OXTR* | *31* | *64* | *VCPKMT* | *59* | *36* |
| *GYS1* | *49* | *46* | *PKP2* | *48* | *47* |  | | |
| *HFE* | *44* | *51* | *PKP4* | *23* | *72* |  | | |
